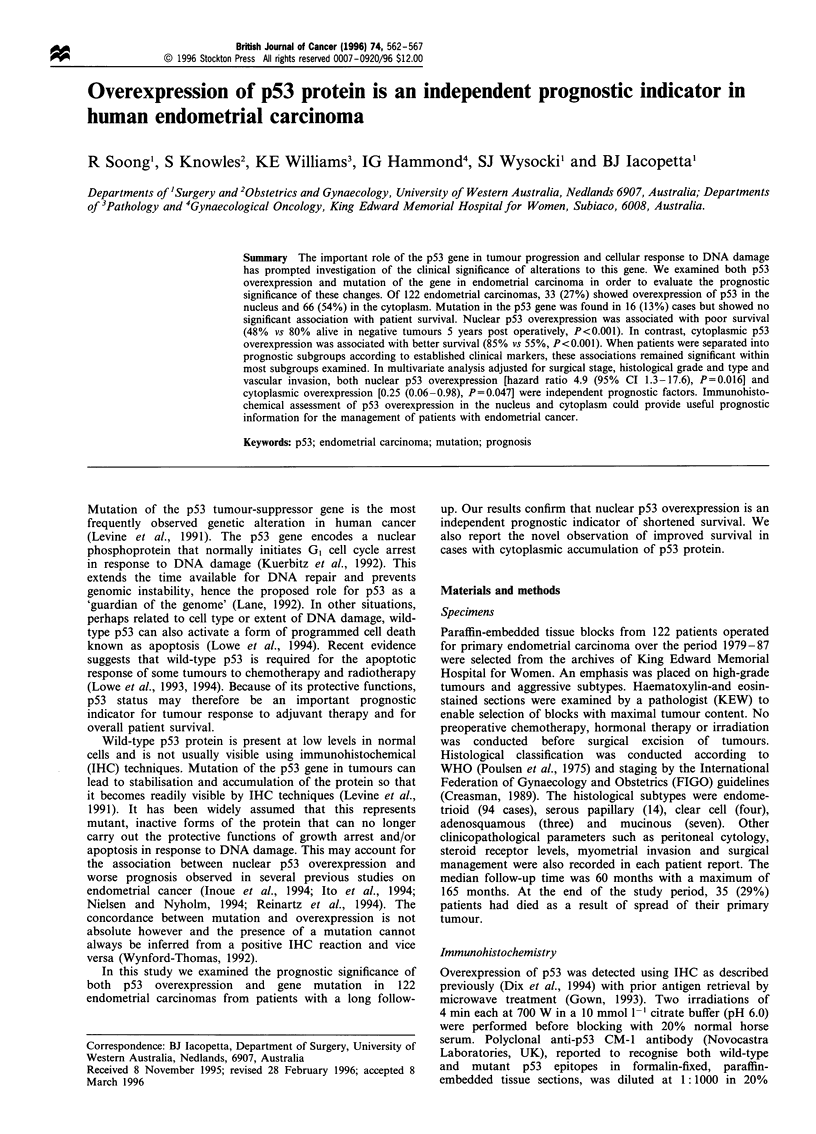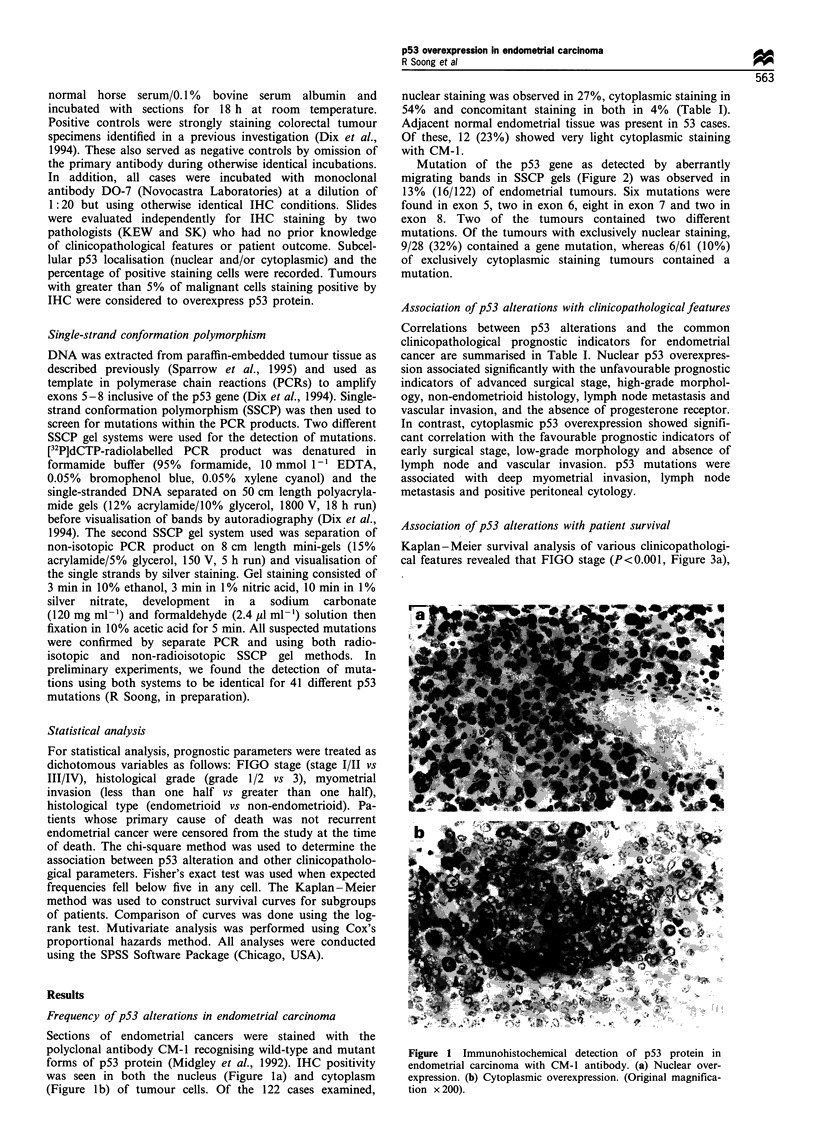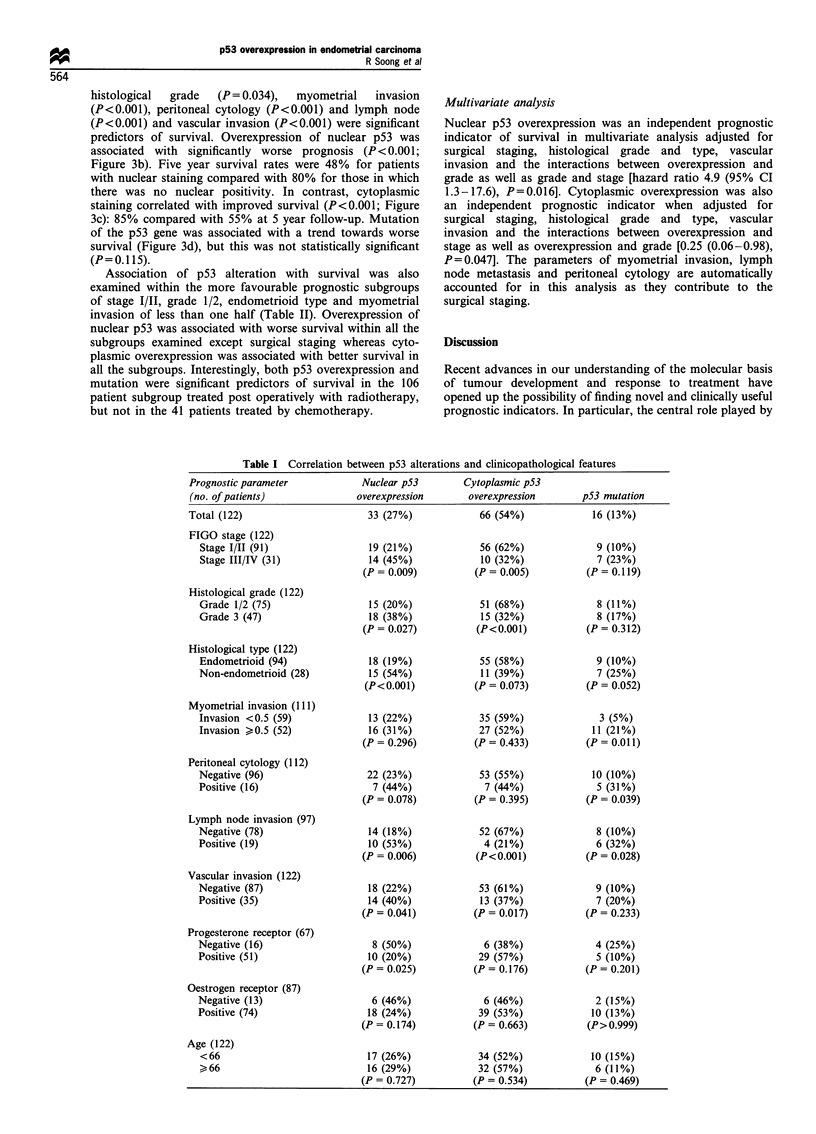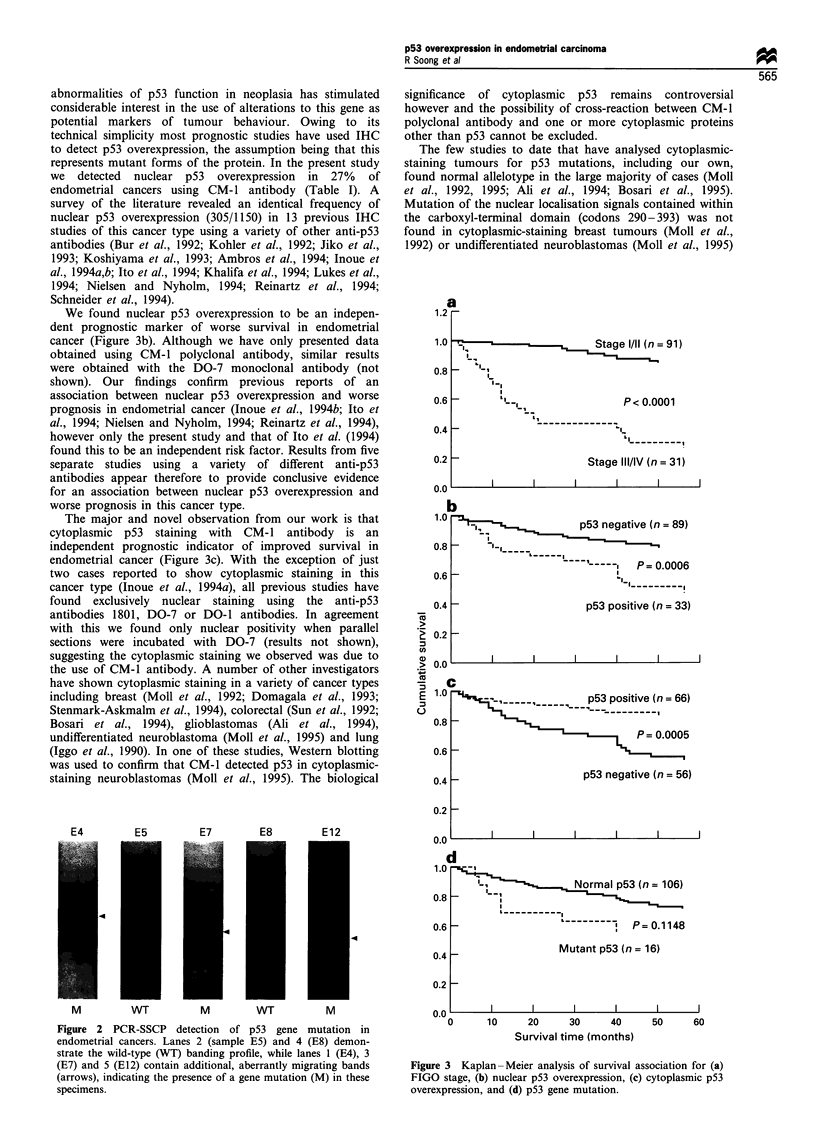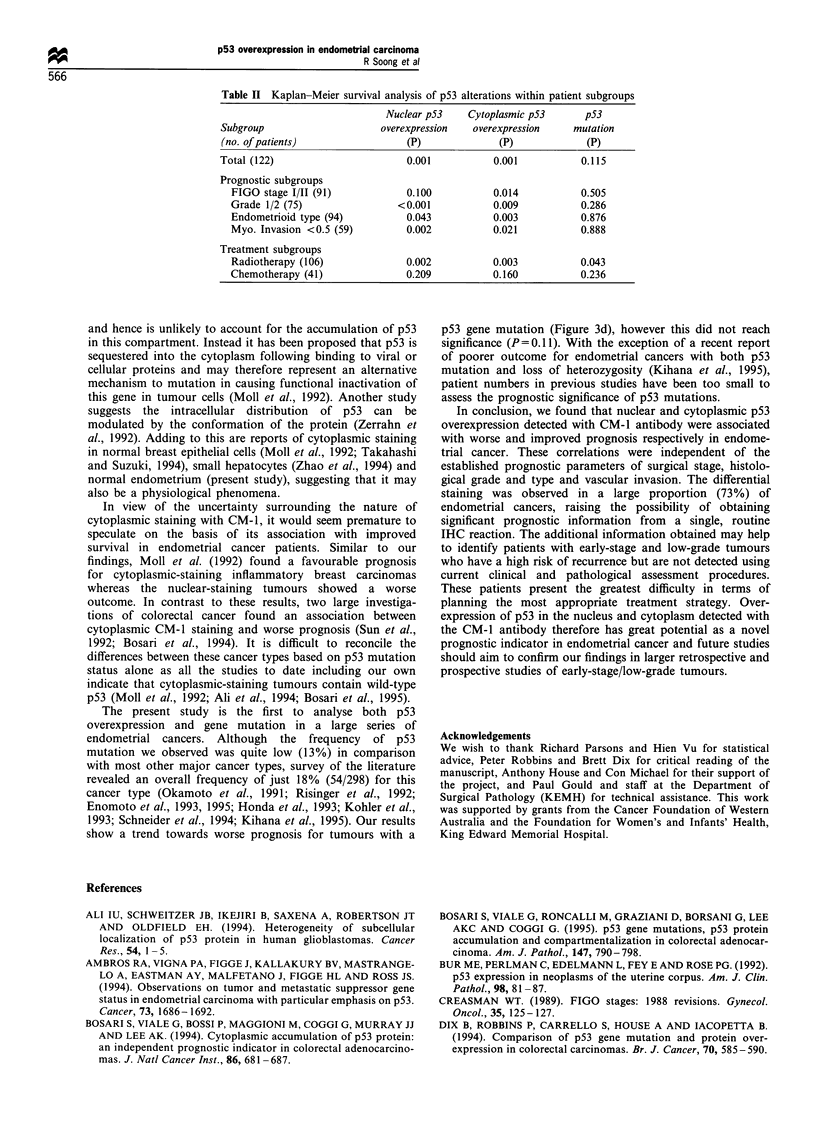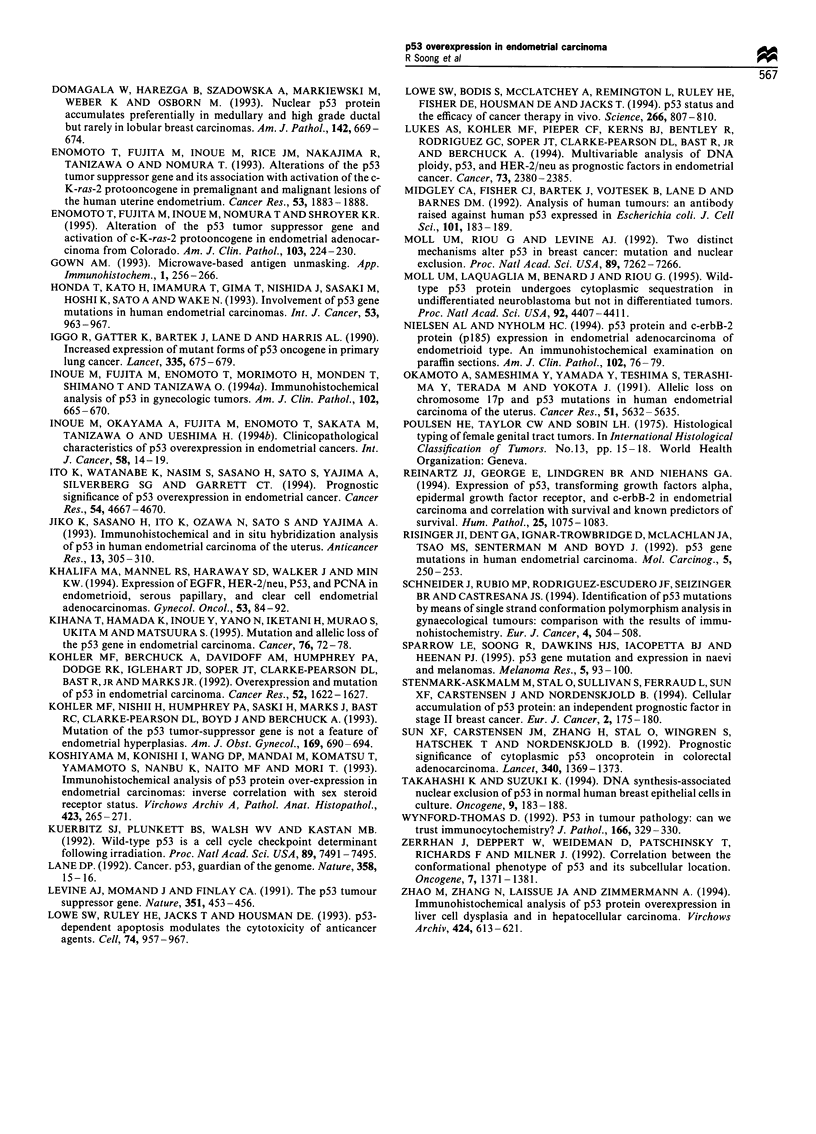# Overexpression of p53 protein is an independent prognostic indicator in human endometrial carcinoma.

**DOI:** 10.1038/bjc.1996.401

**Published:** 1996-08

**Authors:** R. Soong, S. Knowles, K. E. Williams, I. G. Hammond, S. J. Wysocki, B. J. Iacopetta

**Affiliations:** Department of Surgery, University of Western Australia, Nedlands, Australia.

## Abstract

**Images:**


British Journal of Cancer (1996) 74, 562-567
© 1996 Stockton Press All rights reserved 0007-0920/96 $12.00

### Overexpression of p53 protein is an independent prognostic indicator in human endometrial carcinoma

R Soong[1], S Knowles[2], KE Williams[3], IG Hammond[4], SJ Wysocki[1] and BJ Iacopetta[1]

*Departments of [1]Surgery and [2]Obstetrics and Gynaecology, University of Western Australia, Nedlands 6907, Australia; Departments of [3]Pathology and [4]Gynaecological Oncology, King Edward Memorial Hospital for Women, Subiaco, 6008, Australia.*

**Summary** The important role of the p53 gene in tumour progression and cellular response to DNA damage has prompted investigation of the clinical significance of alterations to this gene. We examined both p53 overexpression and mutation of the gene in endometrial carcinoma in order to evaluate the prognostic significance of these changes. Of 122 endometrial carcinomas, 33 (27%) showed overexpression of p53 in the nucleus and 66 (54%) in the cytoplasm. Mutation in the p53 gene was found in 16 (13%) cases but showed no significant association with patient survival. Nuclear p53 overexpression was associated with poor survival (48% *vs* 80% alive in negative tumours 5 years post operatively, $P<0.001$). In contrast, cytoplasmic p53 overexpression was associated with better survival (85% *vs* 55%, $P<0.001$). When patients were separated into prognostic subgroups according to established clinical markers, these associations remained significant within most subgroups examined. In multivariate analysis adjusted for surgical stage, histological grade and type and vascular invasion, both nuclear p53 overexpression [hazard ratio 4.9 (95% CI 1.3-17.6), $P=0.016$] and cytoplasmic overexpression [0.25 (0.06-0.98), $P=0.047$] were independent prognostic factors. Immunohistochemical assessment of p53 overexpression in the nucleus and cytoplasm could provide useful prognostic information for the management of patients with endometrial cancer.

**Keywords:** p53; endometrial carcinoma; mutation; prognosis

Mutation of the p53 tumour-suppressor gene is the most frequently observed genetic alteration in human cancer (Levine *et al.*, 1991). The p53 gene encodes a nuclear phosphoprotein that normally initiates $G_1$ cell cycle arrest in response to DNA damage (Kuerbitz *et al.*, 1992). This extends the time available for DNA repair and prevents genomic instability, hence the proposed role for p53 as a 'guardian of the genome' (Lane, 1992). In other situations, perhaps related to cell type or extent of DNA damage, wild-type p53 can also activate a form of programmed cell death known as apoptosis (Lowe *et al.*, 1994). Recent evidence suggests that wild-type p53 is required for the apoptotic response of some tumours to chemotherapy and radiotherapy (Lowe *et al.*, 1993, 1994). Because of its protective functions, p53 status may therefore be an important prognostic indicator for tumour response to adjuvant therapy and for overall patient survival.

Wild-type p53 protein is present at low levels in normal cells and is not usually visible using immunohistochemical (IHC) techniques. Mutation of the p53 gene in tumours can lead to stabilisation and accumulation of the protein so that it becomes readily visible by IHC techniques (Levine *et al.*, 1991). It has been widely assumed that this represents mutant, inactive forms of the protein that can no longer carry out the protective functions of growth arrest and/or apoptosis in response to DNA damage. This may account for the association between nuclear p53 overexpression and worse prognosis observed in several previous studies on endometrial cancer (Inoue *et al.*, 1994; Ito *et al.*, 1994; Nielsen and Nyholm, 1994; Reinartz *et al.*, 1994). The concordance between mutation and overexpression is not absolute however and the presence of a mutation cannot always be inferred from a positive IHC reaction and vice versa (Wynford-Thomas, 1992).

In this study we examined the prognostic significance of both p53 overexpression and gene mutation in 122 endometrial carcinomas from patients with a long follow-up. Our results confirm that nuclear p53 overexpression is an independent prognostic indicator of shortened survival. We also report the novel observation of improved survival in cases with cytoplasmic accumulation of p53 protein.

Correspondence: BJ Iacopetta, Department of Surgery, University of Western Australia, Nedlands, 6907, Australia
Received 8 November 1995; revised 28 February 1996; accepted 8 March 1996

### Materials and methods

### Specimens

Paraffin-embedded tissue blocks from 122 patients operated for primary endometrial carcinoma over the period 1979-87 were selected from the archives of King Edward Memorial Hospital for Women. An emphasis was placed on high-grade tumours and aggressive subtypes. Haematoxylin-and eosin-stained sections were examined by a pathologist (KEW) to enable selection of blocks with maximal tumour content. No preoperative chemotherapy, hormonal therapy or irradiation was conducted before surgical excision of tumours. Histological classification was conducted according to WHO (Poulsen *et al.*, 1975) and staging by the International Federation of Gynaecology and Obstetrics (FIGO) guidelines (Creasman, 1989). The histological subtypes were endometrioid (94 cases), serous papillary (14), clear cell (four), adenosquamous (three) and mucinous (seven). Other clinicopathological parameters such as peritoneal cytology, steroid receptor levels, myometrial invasion and surgical management were also recorded in each patient report. The median follow-up time was 60 months with a maximum of 165 months. At the end of the study period, 35 (29%) patients had died as a result of spread of their primary tumour.

### Immunohistochemistry

Overexpression of p53 was detected using IHC as described previously (Dix *et al.*, 1994) with prior antigen retrieval by microwave treatment (Gown, 1993). Two irradiations of 4 min each at 700 W in a 10 mmol $l^{-1}$ citrate buffer (pH 6.0) were performed before blocking with 20% normal horse serum. Polyclonal anti-p53 CM-1 antibody (Novocastra Laboratories, UK), reported to recognise both wild-type and mutant p53 epitopes in formalin-fixed, paraffin-embedded tissue sections, was diluted at 1:1000 in 20%

normal horse serum/0.1% bovine serum albumin and incubated with sections for 18 h at room temperature. Positive controls were strongly staining colorectal tumour specimens identified in a previous investigation (Dix *et al.*, 1994). These also served as negative controls by omission of the primary antibody during otherwise identical incubations. In addition, all cases were incubated with monoclonal antibody DO-7 (Novocastra Laboratories) at a dilution of 1:20 but using otherwise identical IHC conditions. Slides were evaluated independently for IHC staining by two pathologists (KEW and SK) who had no prior knowledge of clinicopathological features or patient outcome. Subcellular p53 localisation (nuclear and/or cytoplasmic) and the percentage of positive staining cells were recorded. Tumours with greater than 5% of malignant cells staining positive by IHC were considered to overexpress p53 protein.

*Single-strand conformation polymorphism*

DNA was extracted from paraffin-embedded tumour tissue as described previously (Sparrow *et al.*, 1995) and used as template in polymerase chain reactions (PCRs) to amplify exons 5–8 inclusive of the p53 gene (Dix *et al.*, 1994). Single-strand conformation polymorphism (SSCP) was then used to screen for mutations within the PCR products. Two different SSCP gel systems were used for the detection of mutations. [$^{32}$P]dCTP-radiolabelled PCR product was denatured in formamide buffer (95% formamide, 10 mmol $l^{-1}$ EDTA, 0.05% bromophenol blue, 0.05% xylene cyanol) and the single-stranded DNA separated on 50 cm length polyacrylamide gels (12% acrylamide/10% glycerol, 1800 V, 18 h run) before visualisation of bands by autoradiography (Dix *et al.*, 1994). The second SSCP gel system used was separation of non-isotopic PCR product on 8 cm length mini-gels (15% acrylamide/5% glycerol, 150 V, 5 h run) and visualisation of the single strands by silver staining. Gel staining consisted of 3 min in 10% ethanol, 3 min in 1% nitric acid, 10 min in 1% silver nitrate, development in a sodium carbonate (120 mg $ml^{-1}$) and formaldehyde (2.4 $\mu l$ $ml^{-1}$) solution then fixation in 10% acetic acid for 5 min. All suspected mutations were confirmed by separate PCR and using both radioisotopic and non-radioisotopic SSCP gel methods. In preliminary experiments, we found the detection of mutations using both systems to be identical for 41 different p53 mutations (R Soong, in preparation).

*Statistical analysis*

For statistical analysis, prognostic parameters were treated as dichotomous variables as follows: FIGO stage (stage I/II *vs* III/IV), histological grade (grade 1/2 *vs* 3), myometrial invasion (less than one half *vs* greater than one half), histological type (endometrioid *vs* non-endometrioid). Patients whose primary cause of death was not recurrent endometrial cancer were censored from the study at the time of death. The chi-square method was used to determine the association between p53 alteration and other clinicopathological parameters. Fisher's exact test was used when expected frequencies fell below five in any cell. The Kaplan–Meier method was used to construct survival curves for subgroups of patients. Comparison of curves was done using the log-rank test. Mutivariate analysis was performed using Cox's proportional hazards method. All analyses were conducted using the SPSS Software Package (Chicago, USA).

### Results

*Frequency of p53 alterations in endometrial carcinoma*

Sections of endometrial cancers were stained with the polyclonal antibody CM-1 recognising wild-type and mutant forms of p53 protein (Midgley *et al.*, 1992). IHC positivity was seen in both the nucleus (Figure 1a) and cytoplasm (Figure 1b) of tumour cells. Of the 122 cases examined, nuclear staining was observed in 27%, cytoplasmic staining in 54% and concomitant staining in both in 4% (Table I). Adjacent normal endometrial tissue was present in 53 cases. Of these, 12 (23%) showed very light cytoplasmic staining with CM-1.

Mutation of the p53 gene as detected by aberrantly migrating bands in SSCP gels (Figure 2) was observed in 13% (16/122) of endometrial tumours. Six mutations were found in exon 5, two in exon 6, eight in exon 7 and two in exon 8. Two of the tumours contained two different mutations. Of the tumours with exclusively nuclear staining, 9/28 (32%) contained a gene mutation, whereas 6/61 (10%) of exclusively cytoplasmic staining tumours contained a mutation.

*Association of p53 alterations with clinicopathological features*

Correlations between p53 alterations and the common clinicopathological prognostic indicators for endometrial cancer are summarised in Table I. Nuclear p53 overexpression associated significantly with the unfavourable prognostic indicators of advanced surgical stage, high-grade morphology, non-endometrioid histology, lymph node metastasis and vascular invasion, and the absence of progesterone receptor. In contrast, cytoplasmic p53 overexpression showed significant correlation with the favourable prognostic indicators of early surgical stage, low-grade morphology and absence of lymph node and vascular invasion. p53 mutations were associated with deep myometrial invasion, lymph node metastasis and positive peritoneal cytology.

*Association of p53 alterations with patient survival*

Kaplan–Meier survival analysis of various clinicopathological features revealed that FIGO stage ($P<0.001$, Figure 3a),

**Figure 1** Immunohistochemical detection of p53 protein in endometrial carcinoma with CM-1 antibody. (**a**) Nuclear overexpression. (**b**) Cytoplasmic overexpression. (Original magnification ×200).

histological grade ($P=0.034$), myometrial invasion ($P<0.001$), peritoneal cytology ($P<0.001$) and lymph node ($P<0.001$) and vascular invasion ($P<0.001$) were significant predictors of survival. Overexpression of nuclear p53 was associated with significantly worse prognosis ($P<0.001$; Figure 3b). Five year survival rates were 48% for patients with nuclear staining compared with 80% for those in which there was no nuclear positivity. In contrast, cytoplasmic staining correlated with improved survival ($P<0.001$; Figure 3c): 85% compared with 55% at 5 year follow-up. Mutation of the p53 gene was associated with a trend towards worse survival (Figure 3d), but this was not statistically significant ($P=0.115$).

Association of p53 alteration with survival was also examined within the more favourable prognostic subgroups of stage I/II, grade 1/2, endometrioid type and myometrial invasion of less than one half (Table II). Overexpression of nuclear p53 was associated with worse survival within all the subgroups examined except surgical staging whereas cytoplasmic overexpression was associated with better survival in all the subgroups. Interestingly, both p53 overexpression and mutation were significant predictors of survival in the 106 patient subgroup treated post operatively with radiotherapy, but not in the 41 patients treated by chemotherapy.

### *Multivariate analysis*

Nuclear p53 overexpression was an independent prognostic indicator of survival in multivariate analysis adjusted for surgical staging, histological grade and type, vascular invasion and the interactions between overexpression and grade as well as grade and stage [hazard ratio 4.9 (95% CI 1.3–17.6), $P=0.016$]. Cytoplasmic overexpression was also an independent prognostic indicator when adjusted for surgical staging, histological grade and type, vascular invasion and the interactions between overexpression and stage as well as overexpression and grade [0.25 (0.06–0.98), $P=0.047$]. The parameters of myometrial invasion, lymph node metastasis and peritoneal cytology are automatically accounted for in this analysis as they contribute to the surgical staging.

### Discussion

Recent advances in our understanding of the molecular basis of tumour development and response to treatment have opened up the possibility of finding novel and clinically useful prognostic indicators. In particular, the central role played by

**Table I** Correlation between p53 alterations and clinicopathological features

| *Prognostic parameter (no. of patients)* | *Nuclear p53 overexpression* | *Cytoplasmic p53 overexpression* | *p53 mutation* |
|---|---|---|---|
| Total (122) | 33 (27%) | 66 (54%) | 16 (13%) |
| FIGO stage (122) | | | |
| Stage I/II (91) | 19 (21%) | 56 (62%) | 9 (10%) |
| Stage III/IV (31) | 14 (45%) | 10 (32%) | 7 (23%) |
| | ($P$ = 0.009) | ($P$ = 0.005) | ($P$ = 0.119) |
| Histological grade (122) | | | |
| Grade 1/2 (75) | 15 (20%) | 51 (68%) | 8 (11%) |
| Grade 3 (47) | 18 (38%) | 15 (32%) | 8 (17%) |
| | ($P$ = 0.027) | ($P$<0.001) | ($P$ = 0.312) |
| Histological type (122) | | | |
| Endometrioid (94) | 18 (19%) | 55 (58%) | 9 (10%) |
| Non-endometrioid (28) | 15 (54%) | 11 (39%) | 7 (25%) |
| | ($P$<0.001) | ($P$ = 0.073) | ($P$ = 0.052) |
| Myometrial invasion (111) | | | |
| Invasion <0.5 (59) | 13 (22%) | 35 (59%) | 3 (5%) |
| Invasion ⩾0.5 (52) | 16 (31%) | 27 (52%) | 11 (21%) |
| | ($P$ = 0.296) | ($P$ = 0.433) | ($P$ = 0.011) |
| Peritoneal cytology (112) | | | |
| Negative (96) | 22 (23%) | 53 (55%) | 10 (10%) |
| Positive (16) | 7 (44%) | 7 (44%) | 5 (31%) |
| | ($P$ = 0.078) | ($P$ = 0.395) | ($P$ = 0.039) |
| Lymph node invasion (97) | | | |
| Negative (78) | 14 (18%) | 52 (67%) | 8 (10%) |
| Positive (19) | 10 (53%) | 4 (21%) | 6 (32%) |
| | ($P$ = 0.006) | ($P$<0.001) | ($P$ = 0.028) |
| Vascular invasion (122) | | | |
| Negative (87) | 18 (22%) | 53 (61%) | 9 (10%) |
| Positive (35) | 14 (40%) | 13 (37%) | 7 (20%) |
| | ($P$ = 0.041) | ($P$ = 0.017) | ($P$ = 0.233) |
| Progesterone receptor (67) | | | |
| Negative (16) | 8 (50%) | 6 (38%) | 4 (25%) |
| Positive (51) | 10 (20%) | 29 (57%) | 5 (10%) |
| | ($P$ = 0.025) | ($P$ = 0.176) | ($P$ = 0.201) |
| Oestrogen receptor (87) | | | |
| Negative (13) | 6 (46%) | 6 (46%) | 2 (15%) |
| Positive (74) | 18 (24%) | 39 (53%) | 10 (13%) |
| | ($P$ = 0.174) | ($P$ = 0.663) | ($P$>0.999) |
| Age (122) | | | |
| <66 | 17 (26%) | 34 (52%) | 10 (15%) |
| ⩾66 | 16 (29%) | 32 (57%) | 6 (11%) |
| | ($P$ = 0.727) | ($P$ = 0.534) | ($P$ = 0.469) |

abnormalities of p53 function in neoplasia has stimulated considerable interest in the use of alterations to this gene as potential markers of tumour behaviour. Owing to its technical simplicity most prognostic studies have used IHC to detect p53 overexpression, the assumption being that this represents mutant forms of the protein. In the present study we detected nuclear p53 overexpression in 27% of endometrial cancers using CM-1 antibody (Table I). A survey of the literature revealed an identical frequency of nuclear p53 overexpression (305/1150) in 13 previous IHC studies of this cancer type using a variety of other anti-p53 antibodies (Bur *et al.*, 1992; Kohler *et al.*, 1992; Jiko *et al.*, 1993; Koshiyama *et al.*, 1993; Ambros *et al.*, 1994; Inoue *et al.*, 1994*a*,*b*; Ito *et al.*, 1994; Khalifa *et al.*, 1994; Lukes *et al.*, 1994; Nielsen and Nyholm, 1994; Reinartz *et al.*, 1994; Schneider *et al.*, 1994).

We found nuclear p53 overexpression to be an independent prognostic marker of worse survival in endometrial cancer (Figure 3b). Although we have only presented data obtained using CM-1 polyclonal antibody, similar results were obtained with the DO-7 monoclonal antibody (not shown). Our findings confirm previous reports of an association between nuclear p53 overexpression and worse prognosis in endometrial cancer (Inoue *et al.*, 1994*b*; Ito *et al.*, 1994; Nielsen and Nyholm, 1994; Reinartz *et al.*, 1994), however only the present study and that of Ito *et al.* (1994) found this to be an independent risk factor. Results from five separate studies using a variety of different anti-p53 antibodies appear therefore to provide conclusive evidence for an association between nuclear p53 overexpression and worse prognosis in this cancer type.

The major and novel observation from our work is that cytoplasmic p53 staining with CM-1 antibody is an independent prognostic indicator of improved survival in endometrial cancer (Figure 3c). With the exception of just two cases reported to show cytoplasmic staining in this cancer type (Inoue *et al.*, 1994*a*), all previous studies have found exclusively nuclear staining using the anti-p53 antibodies 1801, DO-7 or DO-1 antibodies. In agreement with this we found only nuclear positivity when parallel sections were incubated with DO-7 (results not shown), suggesting the cytoplasmic staining we observed was due to the use of CM-1 antibody. A number of other investigators have shown cytoplasmic staining in a variety of cancer types including breast (Moll *et al.*, 1992; Domagala *et al.*, 1993; Stenmark-Askmalm *et al.*, 1994), colorectal (Sun *et al.*, 1992; Bosari *et al.*, 1994), glioblastomas (Ali *et al.*, 1994), undifferentiated neuroblastoma (Moll *et al.*, 1995) and lung (Iggo *et al.*, 1990). In one of these studies, Western blotting was used to confirm that CM-1 detected p53 in cytoplasmic-staining neuroblastomas (Moll *et al.*, 1995). The biological significance of cytoplasmic p53 remains controversial however and the possibility of cross-reaction between CM-1 polyclonal antibody and one or more cytoplasmic proteins other than p53 cannot be excluded.

The few studies to date that have analysed cytoplasmic-staining tumours for p53 mutations, including our own, found normal allelotype in the large majority of cases (Moll *et al.*, 1992, 1995; Ali *et al.*, 1994; Bosari *et al.*, 1995). Mutation of the nuclear localisation signals contained within the carboxyl-terminal domain (codons 290–393) was not found in cytoplasmic-staining breast tumours (Moll *et al.*, 1992) or undifferentiated neuroblastomas (Moll *et al.*, 1995)

**Figure 2** PCR-SSCP detection of p53 gene mutation in endometrial cancers. Lanes 2 (sample E5) and 4 (E8) demonstrate the wild-type (WT) banding profile, while lanes 1 (E4), 3 (E7) and 5 (E12) contain additional, aberrantly migrating bands (arrows), indicating the presence of a gene mutation (M) in these specimens.

**Figure 3** Kaplan–Meier analysis of survival association for (**a**) FIGO stage, (**b**) nuclear p53 overexpression, (**c**) cytoplasmic p53 overexpression, and (**d**) p53 gene mutation.

**Table II** Kaplan–Meier survival analysis of p53 alterations within patient subgroups

| *Subgroup (no. of patients)* | *Nuclear p53 overexpression* (P) | *Cytoplasmic p53 overexpression* (P) | *p53 mutation* (P) |
|---|---|---|---|
| Total (122) | 0.001 | 0.001 | 0.115 |
| Prognostic subgroups | | | |
| FIGO stage I/II (91) | 0.100 | 0.014 | 0.505 |
| Grade 1/2 (75) | <0.001 | 0.009 | 0.286 |
| Endometrioid type (94) | 0.043 | 0.003 | 0.876 |
| Myo. Invasion <0.5 (59) | 0.002 | 0.021 | 0.888 |
| Treatment subgroups | | | |
| Radiotherapy (106) | 0.002 | 0.003 | 0.043 |
| Chemotherapy (41) | 0.209 | 0.160 | 0.236 |

and hence is unlikely to account for the accumulation of p53 in this compartment. Instead it has been proposed that p53 is sequestered into the cytoplasm following binding to viral or cellular proteins and may therefore represent an alternative mechanism to mutation in causing functional inactivation of this gene in tumour cells (Moll *et al.*, 1992). Another study suggests the intracellular distribution of p53 can be modulated by the conformation of the protein (Zerrahn *et al.*, 1992). Adding to this are reports of cytoplasmic staining in normal breast epithelial cells (Moll *et al.*, 1992; Takahashi and Suzuki, 1994), small hepatocytes (Zhao *et al.*, 1994) and normal endometrium (present study), suggesting that it may also be a physiological phenomena.

In view of the uncertainty surrounding the nature of cytoplasmic staining with CM-1, it would seem premature to speculate on the basis of its association with improved survival in endometrial cancer patients. Similar to our findings, Moll *et al.* (1992) found a favourable prognosis for cytoplasmic-staining inflammatory breast carcinomas whereas the nuclear-staining tumours showed a worse outcome. In contrast to these results, two large investigations of colorectal cancer found an association between cytoplasmic CM-1 staining and worse prognosis (Sun *et al.*, 1992; Bosari *et al.*, 1994). It is difficult to reconcile the differences between these cancer types based on p53 mutation status alone as all the studies to date including our own indicate that cytoplasmic-staining tumours contain wild-type p53 (Moll *et al.*, 1992; Ali *et al.*, 1994; Bosari *et al.*, 1995).

The present study is the first to analyse both p53 overexpression and gene mutation in a large series of endometrial cancers. Although the frequency of p53 mutation we observed was quite low (13%) in comparison with most other major cancer types, survey of the literature revealed an overall frequency of just 18% (54/298) for this cancer type (Okamoto *et al.*, 1991; Risinger *et al.*, 1992; Enomoto *et al.*, 1993, 1995; Honda *et al.*, 1993; Kohler *et al.*, 1993; Schneider *et al.*, 1994; Kihana *et al.*, 1995). Our results show a trend towards worse prognosis for tumours with a p53 gene mutation (Figure 3d), however this did not reach significance ($P = 0.11$). With the exception of a recent report of poorer outcome for endometrial cancers with both p53 mutation and loss of heterozygosity (Kihana *et al.*, 1995), patient numbers in previous studies have been too small to assess the prognostic significance of p53 mutations.

In conclusion, we found that nuclear and cytoplasmic p53 overexpression detected with CM-1 antibody were associated with worse and improved prognosis respectively in endometrial cancer. These correlations were independent of the established prognostic parameters of surgical stage, histological grade and type and vascular invasion. The differential staining was observed in a large proportion (73%) of endometrial cancers, raising the possibility of obtaining significant prognostic information from a single, routine IHC reaction. The additional information obtained may help to identify patients with early-stage and low-grade tumours who have a high risk of recurrence but are not detected using current clinical and pathological assessment procedures. These patients present the greatest difficulty in terms of planning the most appropriate treatment strategy. Overexpression of p53 in the nucleus and cytoplasm detected with the CM-1 antibody therefore has great potential as a novel prognostic indicator in endometrial cancer and future studies should aim to confirm our findings in larger retrospective and prospective studies of early-stage/low-grade tumours.

**Acknowledgements**
We wish to thank Richard Parsons and Hien Vu for statistical advice, Peter Robbins and Brett Dix for critical reading of the manuscript, Anthony House and Con Michael for their support of the project, and Paul Gould and staff at the Department of Surgical Pathology (KEMH) for technical assistance. This work was supported by grants from the Cancer Foundation of Western Australia and the Foundation for Women's and Infants' Health, King Edward Memorial Hospital.

### References

ALI IU, SCHWEITZER JB, IKEJIRI B, SAXENA A, ROBERTSON JT AND OLDFIELD EH. (1994). Heterogeneity of subcellular localization of p53 protein in human glioblastomas. *Cancer Res.*, **54,** 1–5.

AMBROS RA, VIGNA PA, FIGGE J, KALLAKURY BV, MASTRANGELO A, EASTMAN AY, MALFETANO J, FIGGE HL AND ROSS JS. (1994). Observations on tumor and metastatic suppressor gene status in endometrial carcinoma with particular emphasis on p53. *Cancer*, **73,** 1686–1692.

BOSARI S, VIALE G, BOSSI P, MAGGIONI M, COGGI G, MURRAY JJ AND LEE AK. (1994). Cytoplasmic accumulation of p53 protein: an independent prognostic indicator in colorectal adenocarcinomas. *J. Natl Cancer Inst.*, **86,** 681–687.

BOSARI S, VIALE G, RONCALLI M, GRAZIANI D, BORSANI G, LEE AKC AND COGGI G. (1995). p53 gene mutations, p53 protein accumulation and compartmentalization in colorectal adenocarcinoma. *Am. J. Pathol.*, **147,** 790–798.

BUR ME, PERLMAN C, EDELMANN L, FEY E AND ROSE PG. (1992). p53 expression in neoplasms of the uterine corpus. *Am. J. Clin. Pathol.*, **98,** 81–87.

CREASMAN WT. (1989). FIGO stages: 1988 revisions. *Gynecol. Oncol.*, **35,** 125–127.

DIX B, ROBBINS P, CARRELLO S, HOUSE A AND IACOPETTA B. (1994). Comparison of p53 gene mutation and protein overexpression in colorectal carcinomas. *Br. J. Cancer*, **70,** 585–590.

DOMAGALA W, HAREZGA B, SZADOWSKA A, MARKIEWSKI M, WEBER K AND OSBORN M. (1993). Nuclear p53 protein accumulates preferentially in medullary and high grade ductal but rarely in lobular breast carcinomas. *Am. J. Pathol.*, **142,** 669–674.

ENOMOTO T, FUJITA M, INOUE M, RICE JM, NAKAJIMA R, TANIZAWA O AND NOMURA T. (1993). Alterations of the p53 tumor suppressor gene and its association with activation of the c-K-*ras*-2 protooncogene in premalignant and malignant lesions of the human uterine endometrium. *Cancer Res.*, **53,** 1883–1888.

ENOMOTO T, FUJITA M, INOUE M, NOMURA T AND SHROYER KR. (1995). Alteration of the p53 tumor suppressor gene and activation of c-K-*ras*-2 protooncogene in endometrial adenocarcinoma from Colorado. *Am. J. Clin. Pathol.*, **103,** 224–230.

GOWN AM. (1993). Microwave-based antigen unmasking. *App. Immunohistochem.*, **1,** 256–266.

HONDA T, KATO H, IMAMURA T, GIMA T, NISHIDA J, SASAKI M, HOSHI K, SATO A AND WAKE N. (1993). Involvement of p53 gene mutations in human endometrial carcinomas. *Int. J. Cancer*, **53,** 963–967.

IGGO R, GATTER K, BARTEK J, LANE D AND HARRIS AL. (1990). Increased expression of mutant forms of p53 oncogene in primary lung cancer. *Lancet*, **335,** 675–679.

INOUE M, FUJITA M, ENOMOTO T, MORIMOTO H, MONDEN T, SHIMANO T AND TANIZAWA O. (1994*a*). Immunohistochemical analysis of p53 in gynecologic tumors. *Am. J. Clin. Pathol.*, **102,** 665–670.

INOUE M, OKAYAMA A, FUJITA M, ENOMOTO T, SAKATA M, TANIZAWA O AND UESHIMA H. (1994*b*). Clinicopathological characteristics of p53 overexpression in endometrial cancers. *Int. J. Cancer*, **58,** 14–19.

ITO K, WATANABE K, NASIM S, SASANO H, SATO S, YAJIMA A, SILVERBERG SG AND GARRETT CT. (1994). Prognostic significance of p53 overexpression in endometrial cancer. *Cancer Res.*, **54,** 4667–4670.

JIKO K, SASANO H, ITO K, OZAWA N, SATO S AND YAJIMA A. (1993). Immunohistochemical and in situ hybridization analysis of p53 in human endometrial carcinoma of the uterus. *Anticancer Res.*, **13,** 305–310.

KHALIFA MA, MANNEL RS, HARAWAY SD, WALKER J AND MIN KW. (1994). Expression of EGFR, HER-2/neu, P53, and PCNA in endometrioid, serous papillary, and clear cell endometrial adenocarcinomas. *Gynecol. Oncol.*, **53,** 84–92.

KIHANA T, HAMADA K, INOUE Y, YANO N, IKETANI H, MURAO S, UKITA M AND MATSUURA S. (1995). Mutation and allelic loss of the p53 gene in endometrial carcinoma. *Cancer*, **76,** 72–78.

KOHLER MF, BERCHUCK A, DAVIDOFF AM, HUMPHREY PA, DODGE RK, IGLEHART JD, SOPER JT, CLARKE-PEARSON DL, BAST R, JR AND MARKS JR. (1992). Overexpression and mutation of p53 in endometrial carcinoma. *Cancer Res.*, **52,** 1622–1627.

KOHLER MF, NISHII H, HUMPHREY PA, SASKI H, MARKS J, BAST RC, CLARKE-PEARSON DL, BOYD J AND BERCHUCK A. (1993). Mutation of the p53 tumor-suppressor gene is not a feature of endometrial hyperplasias. *Am. J. Obst. Gynecol.*, **169,** 690–694.

KOSHIYAMA M, KONISHI I, WANG DP, MANDAI M, KOMATSU T, YAMAMOTO S, NANBU K, NAITO MF AND MORI T. (1993). Immunohistochemical analysis of p53 protein over-expression in endometrial carcinomas: inverse correlation with sex steroid receptor status. *Virchows Archiv A, Pathol. Anat. Histopathol.*, **423,** 265–271.

KUERBITZ SJ, PLUNKETT BS, WALSH WV AND KASTAN MB. (1992). Wild-type p53 is a cell cycle checkpoint determinant following irradiation. *Proc. Natl Acad. Sci. USA*, **89,** 7491–7495.

LANE DP. (1992). Cancer. p53, guardian of the genome. *Nature*, **358,** 15–16.

LEVINE AJ, MOMAND J AND FINLAY CA. (1991). The p53 tumour suppressor gene. *Nature*, **351,** 453–456.

LOWE SW, RULEY HE, JACKS T AND HOUSMAN DE. (1993). p53-dependent apoptosis modulates the cytotoxicity of anticancer agents. *Cell*, **74,** 957–967.

LOWE SW, BODIS S, MCCLATCHEY A, REMINGTON L, RULEY HE, FISHER DE, HOUSMAN DE AND JACKS T. (1994). p53 status and the efficacy of cancer therapy in vivo. *Science*, **266,** 807–810.

LUKES AS, KOHLER MF, PIEPER CF, KERNS BJ, BENTLEY R, RODRIGUEZ GC, SOPER JT, CLARKE-PEARSON DL, BAST R, JR AND BERCHUCK A. (1994). Multivariable analysis of DNA ploidy, p53, and HER-2/neu as prognostic factors in endometrial cancer. *Cancer*, **73,** 2380–2385.

MIDGLEY CA, FISHER CJ, BARTEK J, VOJTESEK B, LANE D AND BARNES DM. (1992). Analysis of human tumours: an antibody raised against human p53 expressed in *Escherichia coli. J. Cell Sci.*, **101,** 183–189.

MOLL UM, RIOU G AND LEVINE AJ. (1992). Two distinct mechanisms alter p53 in breast cancer: mutation and nuclear exclusion. *Proc. Natl Acad. Sci. USA*, **89,** 7262–7266.

MOLL UM, LAQUAGLIA M, BENARD J AND RIOU G. (1995). Wild-type p53 protein undergoes cytoplasmic sequestration in undifferentiated neuroblastoma but not in differentiated tumors. *Proc. Natl Acad. Sci. USA*, **92,** 4407–4411.

NIELSEN AL AND NYHOLM HC. (1994). p53 protein and c-erbB-2 protein (p185) expression in endometrial adenocarcinoma of endometrioid type. An immunohistochemical examination on paraffin sections. *Am. J. Clin. Pathol.*, **102,** 76–79.

OKAMOTO A, SAMESHIMA Y, YAMADA Y, TESHIMA S, TERASHIMA Y, TERADA M AND YOKOTA J. (1991). Allelic loss on chromosome 17p and p53 mutations in human endometrial carcinoma of the uterus. *Cancer Res.*, **51,** 5632–5635.

POULSEN HE, TAYLOR CW AND SOBIN LH. (1975). Histological typing of female genital tract tumors. In *International Histological Classification of Tumors.* No.13, pp. 15–18. World Health Organization: Geneva.

REINARTZ JJ, GEORGE E, LINDGREN BR AND NIEHANS GA. (1994). Expression of p53, transforming growth factors alpha, epidermal growth factor receptor, and c-erbB-2 in endometrial carcinoma and correlation with survival and known predictors of survival. *Hum. Pathol.*, **25,** 1075–1083.

RISINGER JI, DENT GA, IGNAR-TROWBRIDGE D, MCLACHLAN JA, TSAO MS, SENTERMAN M AND BOYD J. (1992). p53 gene mutations in human endometrial carcinoma. *Mol. Carcinog.*, **5,** 250–253.

SCHNEIDER J, RUBIO MP, RODRIGUEZ-ESCUDERO JF, SEIZINGER BR AND CASTRESANA JS. (1994). Identification of p53 mutations by means of single strand conformation polymorphism analysis in gynaecological tumours: comparison with the results of immunohistochemistry. *Eur. J. Cancer*, **4,** 504–508.

SPARROW LE, SOONG R, DAWKINS HJS, IACOPETTA BJ AND HEENAN PJ. (1995). p53 gene mutation and expression in naevi and melanomas. *Melanoma Res.*, **5,** 93–100.

STENMARK-ASKMALM M, STAL O, SULLIVAN S, FERRAUD L, SUN XF, CARSTENSEN J AND NORDENSKJOLD B. (1994). Cellular accumulation of p53 protein: an independent prognostic factor in stage II breast cancer. *Eur. J. Cancer*, **2,** 175–180.

SUN XF, CARSTENSEN JM, ZHANG H, STAL O, WINGREN S, HATSCHEK T AND NORDENSKJOLD B. (1992). Prognostic significance of cytoplasmic p53 oncoprotein in colorectal adenocarcinoma. *Lancet*, **340,** 1369–1373.

TAKAHASHI K AND SUZUKI K. (1994). DNA synthesis-associated nuclear exclusion of p53 in normal human breast epithelial cells in culture. *Oncogene*, **9,** 183–188.

WYNFORD-THOMAS D. (1992). P53 in tumour pathology: can we trust immunocytochemistry? *J. Pathol.*, **166,** 329–330.

ZERRHAN J, DEPPERT W, WEIDEMAN D, PATSCHINSKY T, RICHARDS F AND MILNER J. (1992). Correlation between the conformational phenotype of p53 and its subcellular location. *Oncogene*, **7,** 1371–1381.

ZHAO M, ZHANG N, LAISSUE JA AND ZIMMERMANN A. (1994). Immunohistochemical analysis of p53 protein overexpression in liver cell dysplasia and in hepatocellular carcinoma. *Virchows Archiv*, **424,** 613–621.